# Efficacy of Immune Checkpoint Inhibitors in Non-small-cell Lung Cancer Patients With Different Metastatic Sites: A Systematic Review and Meta-Analysis

**DOI:** 10.3389/fonc.2020.01098

**Published:** 2020-07-09

**Authors:** Kaili Yang, Jiarui Li, Chunmei Bai, Zhao Sun, Lin Zhao

**Affiliations:** Department of Medical Oncology, Peking Union Medical College Hospital, Chinese Academy of Medical Sciences and Peking Union Medical College, Beijing, China

**Keywords:** non-small-cell lung cancer, brain metastasis, liver metastasis, immune checkpoint inhibitor, meta-analysis

## Abstract

**Background:** Organ-specific response patterns reported in previous studies indicate different response toward immune checkpoint inhibitors (ICIs) in non-small-cell lung cancer (NSCLC) patients with different metastatic sites. This study aims to compare the efficacy of ICIs with conventional therapy in NSCLC patients with bone, brain or liver metastases.

**Materials and Methods:** MEDLINE, Embase, and CENTRAL were searched for studies comparing ICIs with conventional therapy in NSCLC patients with bone, brain or liver metastases. The pooled hazard ratio (HR) of overall survival (OS) and progression-free survival (PFS) among included studies was analyzed using the random effects model.

**Results:** Eight studies consisting of 988 NSCLC patients were included, 259 with brain metastases and 729 with liver metastases. No available study with bone metastases information was identified. For patients with brain metastases, ICIs significantly improved their OS (HR, 0.57; *P* = 0.007). For patients with liver metastases, both OS (HR, 0.72; *P* = 0.006), and PFS (HR, 0.72; *P* = 0.004) improvements were observed in the ICI treatment arm. Subgroup analysis was conducted based on target of ICIs and treatment regimen. PD-1 inhibitors could benefit patients with liver or brain metastases on OS and PFS (brain metastases: OS, HR, 0.43; *P* < 0.001; liver metastases: PFS, HR, 0.52; *P* = 0.003; OS, HR, 0.66; *P* = 0.001), while PD-L1 inhibitors could not. Patients with brain metastases could only gain OS improvement from ICIs combined with chemotherapy (HR, 0.41; *P* = 0.001), but for patients with liver metastases, the benefit was detected using ICIs single agent (HR, 0.68; *P* = 0.012) or ICIs combined with chemotherapy plus anti-VEGF therapy (HR, 0.52; *P* = 0.005).

**Conclusion:** ICIs could significantly improve OS in NSCLC patients with brain or liver metastases compared with conventional therapy. Patients with brain metastases could only gain OS benefit from ICIs combined with chemotherapy, while those with liver metastases obtained superior OS from ICIs single agent or ICIs combined with chemotherapy plus anti-VEGF therapy.

## Introduction

Lung cancer is the leading cause of cancer-related mortality, with 2.1 million cases diagnosed and 1.8 million death every year in the world (1). Non-small-cell lung cancer (NSCLC) accounts for ~85% of all cases of lung cancer in the United States (2). Emerging therapeutic approaches have improved the prognosis of patients with NSCLC, the most promising among which is immune checkpoint inhibitor (ICI), based on its efficacy on relieving the immune suppression in the tumor microenvironment (TME) (3). Up to date, several ICIs have been approved as the first-line or second-line therapy for the treatment of metastatic NSCLC (4, 5).

Despite the substantial survival improvement of ICIs, identifying the population who can benefit from immunotherapy is still a challenge. Bone, brain, and liver are among the most frequent metastatic sites in NSCLC, with about 34% bone metastases, 39% nervous system metastases and 20% liver metastases reported in a study investigating more than 20,000 cases (6, 7). In addition, population-based studies suggest metastases to bone, brain, and liver conferred poor prognosis (6, 8). Regarding the great therapeutic efficacy of ICIs, whether patients with different metastatic sites can benefit from ICIs uniformly is being intensively investigated. Difference in survival and response according to metastatic sites was observed in multiple retrospective studies (9, 10). A lower organ-specific response rate to nivolumab was observed in liver metastases compared with metastases to lymph nodes (8% vs. 28%) in a retrospective study (9). In a real-world cohort investigating the efficacy of nivolumab in patients with NSCLC, the presence of liver metastases predicted worse overall survival (4.0 vs. 9.0 months, *p* < 0.001), while pulmonary metastasis conferred a better outcome (8.8 vs. 5.6 months, *p* = 0.004) (10). Among different metastatic sites, bone, brain, and liver metastases were generally regarded as independent poor prognostic factors for ICI therapies (11–14). However, these results did not compare the efficacy of ICIs with other conventional treatments. Considering the relatively high cost and potential immune-related adverse effects of ICIs, the therapeutic choice for NSCLC patients with specific metastases is still a problem to be solved. Several phase 3 clinical trials have reported the efficacy of ICIs compared with chemotherapies in subgroups of NSCLC patients with baseline brain or liver metastases (15, 16). Nevertheless, the results were controversial. Early data from the KEYNOTE-189 study suggested patients with baseline brain metastases benefitting from ICI intervention arm while other studies, for example, KEYNOTE-024, reached the opposite conclusion (15, 16).

Therefore, we conducted this meta-analysis to comprehensively investigate whether NSCLC patients with bone, brain or liver metastases could gain more benefits from ICIs compared with conventional treatments.

## Materials and Methods

### Literature Search and Study Selection

The Preferred Reporting Items for Systematic Review and Meta-analyses (PRISMA) statement was used to perform this systematic review and meta-analysis (17). The protocol was registered on the International Prospective Register of Systematic Reviews (PROSPERO) before conducting this study (ID: CRD42020164348). A comprehensive literature search via MEDLINE, Embase and CENTRAL up to May 20, 2020 was performed by two investigators (JRL and KLY) independently. Keywords for the query term included *Lung Neoplasms, NSCLC, Neoplasm Metastasis, checkpoint inhibitor, CTLA-4, PD-1, PD-L1, ipilimumab, atezolizumab, durvalumab, pembrolizumab, nivolumab* (Supplementary Table 1). References from published studies were also manually scanned to identify additional relevant trials.

Both inclusion and exclusion criteria were prespecified. The inclusion criteria were listed as follows: (1) patients with histologically or cytologically confirmed NSCLC; (2) studies comparing ICIs (single agent or in combination with chemotherapy or targeted therapy) vs. systematic chemotherapy or targeted therapy or combination of both; (3) available clinical outcomes of patients with baseline bone, brain or liver metastases; (4) any perspective or retrospective studies. The primary outcomes were overall survival (OS) and progression-free survival (PFS). Studies with following characteristics were excluded: (1) duplication of previous studies; (2) publication types such as case report, meta-analysis, and review. For studies with multiple publications, the most recent publication was included. Studies were screened independently by two authors (JRL and KLY). Disagreements were solved by consensus or with a third author (LZ) if necessary.

### Data Extraction and Quality Assessment

Data were extracted independently by two authors (JRL and KLY) using a predefined extraction form, including the following information: first author's name, trial name, year of publication, study population, metastatic site, number of patients, intervention, comparison, primary outcomes.

The risk of bias of included studies was independently assessed by two authors (JRL and KLY). Discrepancies were solved by consensus or with a third author (LZ) if necessary. The Cochrane Risk of Bias Tool was used to estimate the quality of randomized controlled trials (RCTs) (18). For retrospective studies or *post-hoc* analysis of subgroups from RCTs, the Newcastle-Ottawa Scale was applied to assess the risk of bias (19). Studies scored ≥ 7 were regarded as being of high quality.

### Statistical Analysis

Efficacy of ICIs on outcomes compared to conventional therapy was measured by hazard ratio (HR) with corresponding 95% confidence interval (CI). The random effects model was used to compute the pooled HR of included studies (20). Cochrane Q test and *I*^2^ test were used to evaluate the heterogeneity among included studies, which was considered statistically significant as *P* < 0.1 or *I*^2^>50%. Subgroup analyses were conducted based on target of ICIs, and treatment regimen of the intervention group. Sensitivity analysis was performed to assess the bias risk of one single study on the pooled result by a leave-one-out approach. Publication bias was evaluated by Begg's and Egger's test.

Stata v15.1 (Stata Corporation, College Station, TX, USA) was applied to perform all statistical analyses. *P*-values were two-sided and considered statistically significant if *P* < 0.05 except for the Cochrane Q test.

## Results

### Eligible Studies and Characteristics

**Figure 1:**
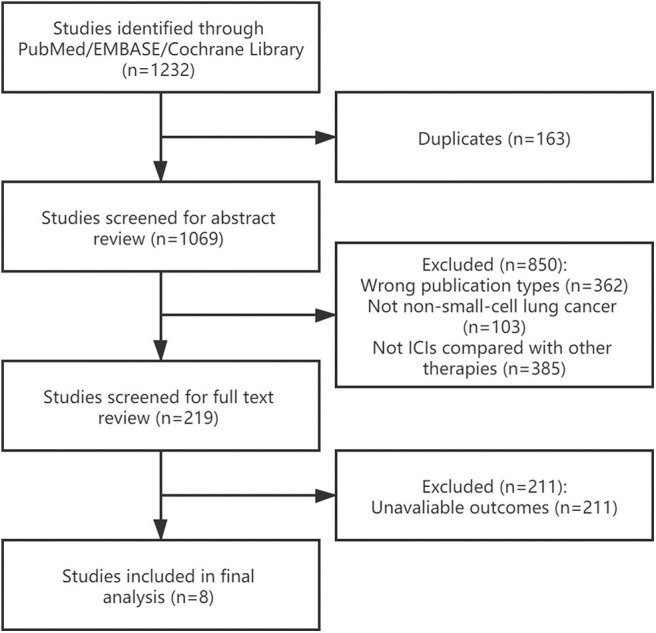
Flowchart diagram of literature search and study selection.

A total of 1,232 studies was initially identified, 163 of which were excluded due to duplications. After screening abstract and full text of references according to the eligible criteria, eight studies were included (15, 21–27). Figure 1 shows the process of study selection.

The main characteristics of included studies were summarized in Table 1. Briefly, 988 cases from eight studies were included, 259 of which with brain metastases, and 729 with liver metastases. No study with available bone metastases information was identified. All the included studies were subgroup analyses of multicenter, randomized, phase 3 trials, published between 2016 and 2019. For metastatic sites, three studies provided OS data of brain metastases (15, 21, 27), while six studies with OS or PFS data of liver metastases (21, 23–27). Two studies included patients who had received 1–2 previous cytotoxic chemotherapy regimens (22, 24), while eligible patients were chemotherapy-naïve in other six studies (15, 21, 23, 25–27). A minimum PD-L1 tumor proportion score of 50% was required in the KEYNOTE-024 study (15), whereas the PD-L1 expression status was not mentioned in other studies. PD-1 inhibitors were applied in three studies (15, 24, 27), while PD-L1 inhibitors were used in 5 studies (21–23, 25, 26). ICI monotherapy were compared with chemotherapy in three studies (15, 22, 24). Four studies applied ICIs combined with chemotherapy vs. chemotherapy alone (21, 25–27), and particularly in one study, ICI was combined with anti-VEGF therapy plus chemotherapy, compared with anti-VEGF therapy plus chemotherapy (23).

**Table 1:** Baseline characteristics of included studies.

**Author**	**Trial name**	**Year**	**Study population**	**No. of baseline liver metastases**	**No. of baseline brain metastases**	**Intervention**	**Comparison**	**Treatment line**	**PD-L1 expression**	**Primary outcomes**	**Quality**
Reck et al. (15)	KEYNOTE-024	2016	Stage IV NSCLC with no sensitizing EGFR mutations or ALK translocations	–	28	Pembrolizumab	Platinum-based chemotherapy	1	>50%	OS	High
Gadgeel et al. (22)	OAK	2019	Squamous or non-squamous NSCLC	–	123	Atezolizumab	docetaxel	≥2	–	OS	High
Jotte et al. (26)	IMpower131	2018	Stage IV squamous NSCLC	139	–	Atezolizumab + carboplatin + nab-paclitaxel	Carboplatin + nab-paclitaxel	≥1 (^*^)	–	PFS	High
Barlesi et al. (21)	IMpower132	2018	Metastatic non-squamous NSCLC lacking sensitizing EGFR or ALK mutations	73	–	Atezolizumab + carboplatin/cisplatin + pemetrexed	Carboplatin/cisplatin + pemetrexed	1	–	OS, PFS	High
Vokes et al. (24)	Checkmate 017 and Checkmate 057	2018	Stage IIIB/IV NSCLC squamous or non-squamous NSCLC	193	–	Nivolumab	Docetaxel	≥2	–	OS	High
West et al. (25)	IMpower130	2019	Stage IV non-squamous NSCLC	100	–	Atezolizumab + carboplatin + nab-paclitaxel	Carboplatin + nab-paclitaxel	≥1 (^*^)	–	OS, PFS	High
Reck et al. (23)	IMpower150	2019	Stage IV metastatic non-squamous NSCLC	109	–	Atezolizumab + bevacizumab + carboplatin + paclitaxel	Bevacizumab + carboplatin + paclitaxel	≥1 (^*^)	–	OS, PFS	High
Garassino et al. (27)	KEYNOTE-189	2019	Metastatic non-squamous NSCLC without sensitizing EGFR or ALK mutations	115	108	Pembrolizumab + platinum-based drug + pemetrexed	Placebo + platinum-based drug + pemetrexed	1	–	OS, PFS	High

*OS, overall survival; PFS, progression-free survival*.

* *eligible patients of this study were chemotherapy-naïve. For patients with a sensitizing mutation in the EGFR gene or ALK fusion oncogene, they must have had disease progression or intolerance to treatment with at least one tyrosine inhibitor*.

The Newcastle-Ottawa Scale was applied to evaluate the risk of bias of included studies. Overall, the methodological quality of all included trials was relatively good (Table 1).

### Effect of ICIs on Patients With Brain Metastases

**Figure 2:**
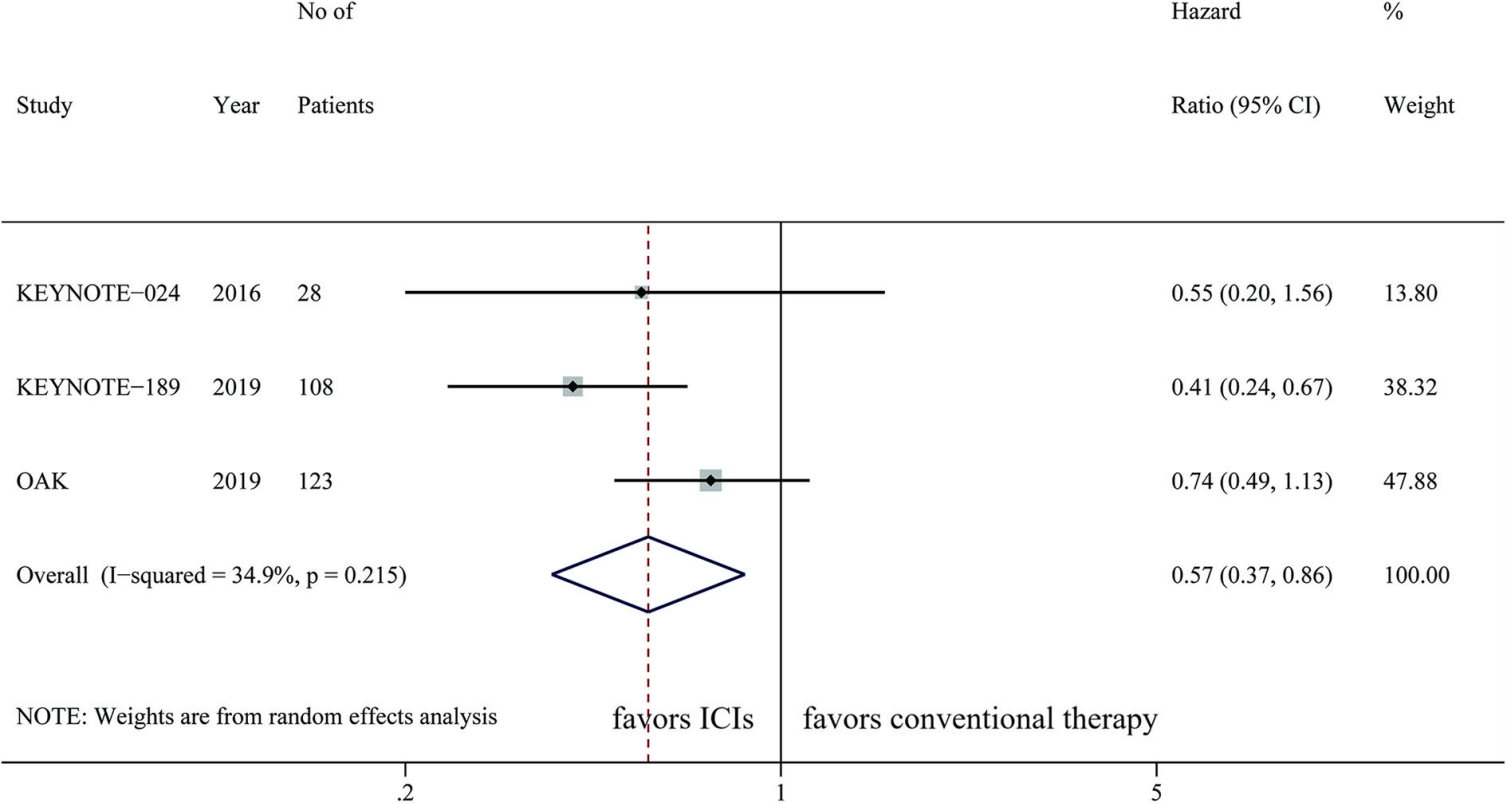
Efficacy of immune checkpoint inhibitors on OS in NSCLC patients with brain metastases.

A total of three studies with 259 cases was integrated to analyze the effect of ICIs on patients with brain metastases, with OS as the primary outcome. Only KEYNOTE-189 evaluated the efficacy of ICIs on PFS, which was not suitable for data synthesis. The pooled result showed that ICIs were significantly correlated with longer OS than chemotherapy (HR, 0.57; 95%CI, 0.37–0.86; *P* = 0.007) with low statistical heterogeneity (*I*^2^=34.9%; *P* = 0.215) (Figure 2). Subgroup analysis showed that patients with brain metastases could benefit more from PD-1 inhibitors than chemotherapy (HR, 0.43; 95%CI, 0.27–0.69; *P* < 0.001). However, PD-L1 inhibitors did not provide significantly longer OS to this population compared with chemotherapy (HR, 0.74; 95%CI, 0.49–1.13; *P* = 0.158) (Table 2). ICI monotherapy did not bring more improvements to patients with brain metastases compared with chemotherapy (HR, 0.71; 95%CI, 0.48–1.04; *P* = 0.082), while ICIs combined with chemotherapy showed a superior OS (HR, 0.41; 95%CI, 0.24–0.67; *P* = 0.001) for this population (Table 2).

**Table 2:** Results of subgroup analysis.

**Group**		**No. of studies**	**Test of association**			**Test of heterogeneity**	
			**HR**	**95% CI**	***P-*Value**	***I*^**2**^ (%)**	***P-*Value**
Brain metastases	**Overall survival**						
	Total	3	0.57	0.37–0.86	0.007	34.9	0.215
	Target of ICIs						
	PD-1	2	0.43	0.27–0.69	<0.001	0	0.616
	PD-L1	1	0.74	0.49–1.13	0.158	–	–
	Treatment regimen						
	ICI monotherapy	2	0.71	0.48–1.04	0.082	0	0.600
	ICI combined with chemotherapy	1	0.41	0.24–0.67	0.001	–	–
Liver metastases	**Overall survival**						
	Total	5	0.72	0.57–0.91	0.006	31.7	0.210
	Target of ICIs						
	PD-1	2	0.66	0.51–0.85	0.001	0	0.742
	PD-L1	3	0.84	0.63–1.12	0.324	26.2	0.258
	Treatment regimen						
	ICI monotherapy	1	0.68	0.50–0.91	0.012	–	–
	ICI combined with chemotherapy	3	0.84	0.63–1.12	0.324	26.2	0.258
	ICI combined with chemotherapy plus anti-VEGF therapy	1	0.52	0.33–0.82	0.005	–	–
	**Progression-free survival**						
	Total	5	0.65	0.49–0.87	0.004	55.7	0.06
	Target of ICIs						
	PD-1	1	0.52	0.34–0.81	0.003	–	–
	PD-L1	4	0.69	0.49–0.97	0.034	61.1	0.052
	Treatment regimen						
	ICI combined with chemotherapy	4	0.73	0.58–0.92	0.008	15.7	0.313
	ICI combined with chemotherapy plus anti-VEGF therapy	1	0.41	0.26–0.62	<0.001	–	–

*HR, hazard ratio; CI, confidence interval; ICI, immune checkpoint inhibitor*.

### Effect of ICIs on Patients With Liver Metastases

**Figure 3:**
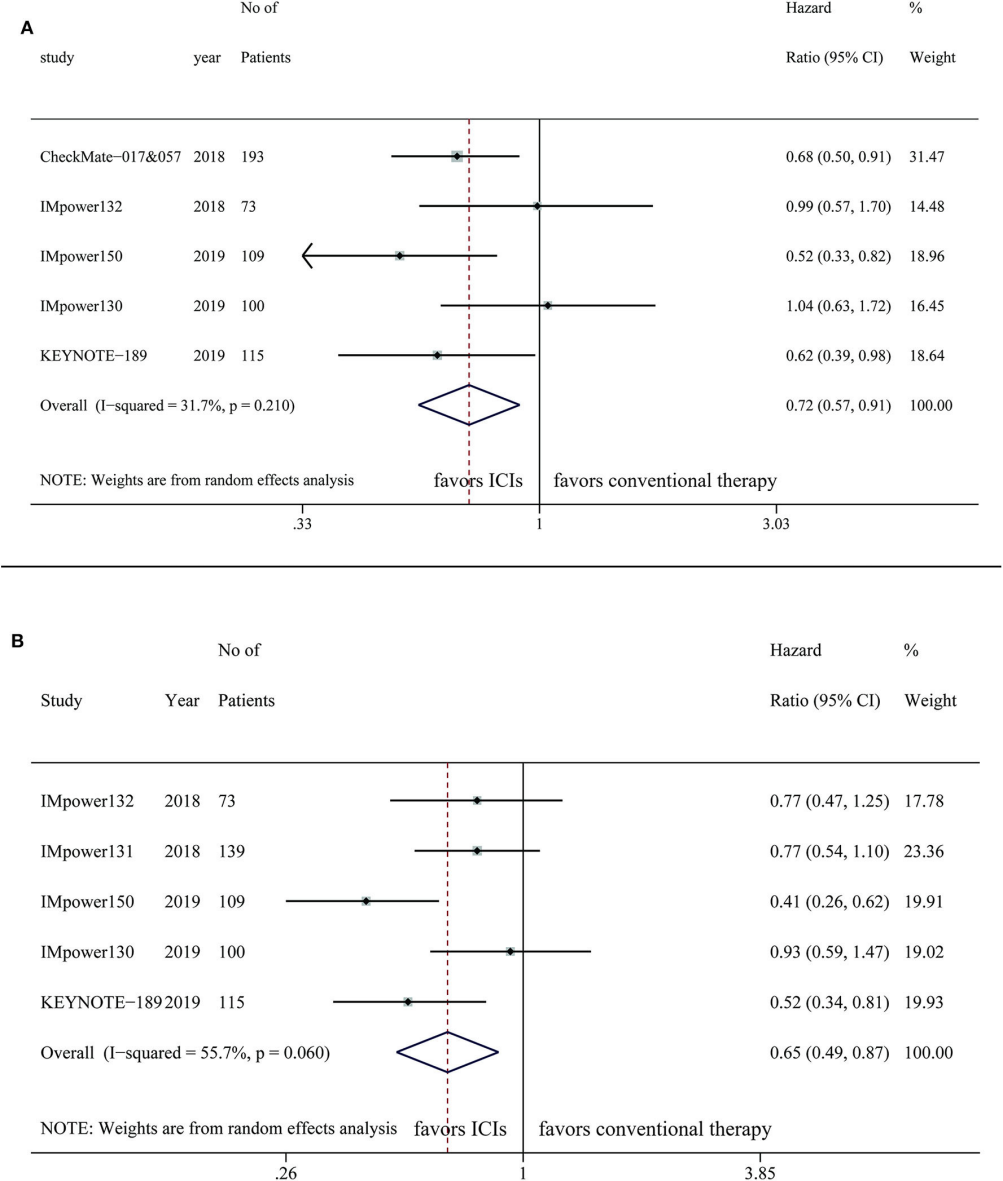
Efficacy of immune checkpoint inhibitors in NSCLC patients with liver metastases on **(A)** OS **(B)** PFS.

Five studies provided OS outcome of 590 NSCLC patients with liver metastases, the pooled result demonstrated a superior OS in the intervention arm (HR, 0.72; 95%CI, 0.57–0.91; *P* = 0.006) with relatively low statistical heterogeneity (*I*^2^=31.7%; *P* = 0.210) (Figure 3A). Benefit of OS in the ICI treatment arm compared with control was observed when PD-1 inhibitors were applied (HR, 0.66; 95%CI, 0.51–0.85; *P* = 0.001), but not for PD-L1 inhibitors (HR, 0.80; 95%CI, 0.51–1.26; *P* = 0.338) (Table 2). Survival improvements were found to be statistically significant when the intervention arm was ICI single agent (HR, 0.68; 95%CI, 0.50–0.91; *P* = 0.012) or ICI combined with chemotherapy plus anti-VEGF therapy (HR, 0.52; 95%CI, 0.33–0.82; *P* = 0.005), but not for ICIs only combined with chemotherapy (HR, 0.84; 95%CI, 0.63–1.12; *P* = 0.324) (Table 2).

Five studies were included for the analysis of PFS of 536 NSCLC patients with liver metastases, indicating patients treated with ICIs have longer PFS than the control group (HR, 0.65; 95%CI, 0.49–0.87; *P* = 0.004) with significant heterogeneity (*I*^2^=55.7%; *P* = 0.06) (Figure 3B). For patients with liver metastases, longer PFS was observed in the ICI arm compared with control, regardless of targets (PD-1: HR, 0.52; 95%CI, 0.34–0.81; *P* = 0.003; PD-L1: HR, 0.69; 95%CI, 0.49–0.97; *P* = 0.034) or the treatment regimen of intervention arm (ICI combined with chemotherapy: HR, 0.73; 95%CI, 0.58–0.92; *P* = 0.008; ICI combined with chemotherapy plus anti-VEGF therapy: HR, 0.41; 95%CI, 0.26–0.62; *P* < 0.001) (Table 2).

### Sensitivity Analysis and Publication Bias

Sensitivity analysis was conducted using the leave-one-out approach to evaluate the effect of each study on the pooled HR. No single study dominates the final interpretation of the pooled result, indicating a relatively good stability (Supplementary Figure 1).

Visual inspection of the Begg funnel plots was symmetry, indicating absence of significant publication bias (Supplementary Figure 2). Further tests suggested no statistically significant publication bias was detected in OS for patients with brain metastases (Begg's test, *P* = 1; Egger's test, *P* = 0.79), OS (Begg's test, *P* = 0.462; Egger's test, *P* = 0.513), and PFS (Begg's test, *P* = 1; Egger's test, *P* = 0.909) for patients with liver metastases.

## Discussion

One of the major challenges of current cancer immunotherapy is understanding organ-specific tumor immune response (28). The TME differs substantially across various organ sites where the tumor evolves, which in turn influences tumor development and host anti-tumor immune response (29). Previous studies have demonstrated organ-specific response patterns to ICI therapy in metastatic NSCLC, indicating the importance of tumor metastatic sites in guiding immunotherapy strategy (9, 30). However, since many studies have reported the effect of metastatic sites on ICI efficacy, no study has been conducted to comprehensively compare the efficacy of ICIs with conventional systematic therapies in regard of metastatic sites.

This systematic review and meta-analysis aimed to compare the efficacy of ICIs with conventional therapies on NSCLC patients with bone, brain or liver metastases. Our study revealed that NSCLC patients with brain metastases could obtain OS improvements from ICI therapy compared with conventional treatment, and for those with liver metastases, they could benefit from ICIs in terms of both OS and PFS. In this meta-analysis, no eligible studies investigating patients with bone metastases were identified. Although previous studies suggested that bone involvement was independent poor prognostic factor for immunotherapy, the relative benefit of ICIs compared with chemotherapy remains obscure. More randomized controlled trials are required to directly elucidate this issue (10, 14).

Brain metastases are normally considered as a frequent metastatic site of advanced NSCLC with unfavorable prognosis (31). Systematic treatments including targeted treatment and chemotherapy are applied to patients without neurological symptoms, with OS ranging from 5 to 16 months (32). Pivotal clinical trials of ICIs generally excluded patients with symptomatic brain metastases, but those with asymptomatic brain metastases were allowed (33). Several recent studies have demonstrated promising efficacy of ICIs in NSCLC patients with brain metastases. Remarkable disease control rate (DCR) of 39% was observed in a cohort of 409 patients with asymptomatic or controlled brain metastases of non-squamous NSCLC (34). A phase 2 trial reported a brain metastases response of 29.7% in patients treated with pembrolizumab with PD-L1 expression of at least 1% (35). However, these studies were single-arm trials without a control group, making it difficult to decide which treatment is superior. Regarding on this issue, our analysis suggests that patients with asymptomatic brain metastases obtain superior OS under the ICI treatment. Both TME and tumor intrinsic features of brain metastases contribute to this efficacy. Evidence showed that the integrity of blood-brain barrier (BBB) was compromised in brain metastases, allowing substantial infiltration of immune suppressive cell types, which may also make it possible for antibodies to cross the BBB and functionate (36). Besides, dense infiltration of lymphocytes was observed in specimens of brain metastases, providing the basis for response to ICIs (37). For tumor cell-inherent factors, high mutation load was observed in brain metastases, which is associated with increased frequency of neoantigens and may contribute to improved response to checkpoint inhibition (38). Only three studies with available baseline brain metastases data was included in this analysis. Therefore, large-scale RCTs are further required to reach the conclusion.

Conventional treatment of liver metastases consists of systematic and palliative therapy (39). With the advent of immunotherapy with revolutionary efficacy, however, several studies have demonstrated liver metastases as an independent poor prognostic factor of immunotherapy for NSCLC (11–13). Patients with liver metastases exhibited significantly shorter OS (mOS, 3.12 months) and PFS (mPFS, 1.35 months) compared with those without liver metastases (mOS, 11.37 months; mPFS, 3.75 months) in a retrospective study, with an overall response rate (ORR) of 22.5% (40). One possible explanation is the immunoregulatory hepatic microenvironment. As a major metabolic organ, liver has unique immunoregulatory functions in order to prevent the induction of immunity against innocuous antigens (41). Local hepatic antigen-presenting cells induce T cell tolerance by multiple mechanisms, including clonal elimination, induction of T cell anergy and recruitment of regulatory T cells, and the presence of hepatic sinusoids provides a large immunoregulatory platform for all the interactions (42). This tolerogenic hepatic microenvironment may interfere response of liver metastases toward ICIs. In NSCLC patients with baseline liver metastases treated with PD-1 inhibitor, decreased marginal CD8+ T cells infiltration was observed, in accordance with lower PFS and objective response rates compared with those without liver metastases (13). Despite all the confirmed mechanisms, however, whether patients with liver metastases obtain longer survival from ICI therapies vs. conventional treatments remains controversial. A previous meta-analysis demonstrated superior OS of chemo-immunotherapy in patients with liver involvement, in which three trials regarding liver metastases were included (43). In our analysis consisting of six trials, consistently, superior OS and PFS were observed in the ICI intervention arm, suggesting a preference of ICIs for the therapeutic decision when regarding NSCLC patients with liver metastases.

Subgroup analysis was conducted to identify possible clinical factors influencing the efficacy of ICIs. In terms of ICI target, patients could gain statistically significant OS and PFS benefit from PD-1 inhibitors regardless of metastatic sites, which was not observed in those anti-PD-L1 therapies. At the moment there is no trial directly comparing the efficacy of PD-1 and PD-L1 inhibitors. Two previous large phase 1 studies have suggested PD-1 inhibitor could achieve higher ORR than PD-L1 inhibitor (20%–25% vs. 6%–17%) in patients with advanced solid tumors including NSCLC (44, 45). Furthermore, a recent meta-analysis using paired clinical trials with similar clinical characteristics was conducted to compare the efficacy between PD-1 and PD-L1 inhibitors, suggesting superior OS and PFS benefits of PD-1 inhibitors (46). One possible explanation is that PD-1 inhibitors can block the interactions between PD-1 and PD-L1, as well as PD-L2, which is not viable for PD-L1 inhibitor (47). PD-L2 expression was also identified as a key prognostic factor of ICI treatment in previous studies, and tumors might achieve immune escape through the PD-1/PD-L2 axis under the insufficient blockage of PD-L1 inhibitors (48).

For the choice of single agent or ICI combined with systematic therapy, whether systematic chemotherapy should be combined with ICI is still under investigation, while results of several studies support this combination. Several clinical trials demonstrated higher ORR in patients treated with combination therapy over ICI single agent (15, 49–51). Besides, a recent meta-analysis showed that chemo-immunotherapy could improve OS and PFS in conditions traditionally thought to be weakly immunogenic (43). As many chemotherapy agents functionalize by damaging DNA structure, they may increase the mutation frequency and neoantigen formation, playing a synergistic role with ICIs and thus increase their efficacy (38). In this analysis, consistently, superior OS was observed ICIs combined with systematic chemotherapy for patients with brain metastases, while the benefit of monotherapy was not statistically significant. This result should be interpreted with caution as only three available studies were included in the analysis. A recent single-arm study has demonstrated clinically meaningful intracranial efficacy of 29.7% in 37 patients treated with pembrolizumab monotherapy (35). We cannot exclude the potential efficacy of ICIs administrated as single agent in patients with brain metastases at present, and the superiority of combination therapy should be validated in larger trials. Currently, several ongoing trials have been investigating the efficacy and safety of ICIs combined with other treatment options in treating patients with brain metastases, such as chemotherapy and radiotherapy (52). We can expect more rigorous evidence for the choice of treatment regimens in the future.

For patients with liver metastases, OS benefit was not observed with ICIs simply combined with chemotherapy, unless the addition of anti-VEGF treatment. Another recent meta-analysis investigating the efficacy of chemotherapy combined with ICIs reached the same conclusion (43). Simple addition of chemotherapy may not act synergistically with ICIs in the context of liver, since cytotoxic chemotherapy also targets proliferating benign cells including immune cells (53). However, the importance of combining anti-VEGF therapy with ICIs should be addressed. VEGF plays an important role in metastatic process to organs with abundant blood supply such as liver. Existing hepatic vessels can be utilized by metastatic cells, and the neovascularization process can be triggered by VEGF, creating the structurally and functionally abnormal tumor vasculature, which in turn facilitates the growth and progression of metastases (54). Bevacizumab-induced tumor vasculature normalization, which promotes T cell infiltration in the TME, may work synergistically with ICI and promotes its antitumor activity (55). Beyond that, in treating NSCLC patients with brain metastases, the application of bevacizumab could also reduce the level of circulating myeloid-derived suppressor cells in peripheral blood, suggesting its potential to induce a more effective anti-tumor microenvironment in metastatic site not just limited to liver (56). Altogether, our study supports ICIs combined with systematic chemotherapy in treating NSCLC patients with brain metastases, and for those with liver metastases, the addition of VEGF blockage to enhance the activity of ICIs is also necessary. It should be noted that based on limited clinical evidence, this suggestion is rather preliminary and exploratory. More prospective large-scale studies are required to further elucidate this problem.

Among other prognostic factors of immunotherapy, PD-L1 expression on tumor or immune cells was the most frequently studied biomarker, and several FDA approvals were linked to a specific PD-L1 threshold (57). This study did not investigate the relationship between PD-L1 expression and efficacy of ICIs in patients with brain or liver metastases, as only the KEYNOTE-024 study mentioned a PD-L1 expression threshold of 50% (15). The predictive value of PD-L1 expression in patients with specific metastases was demonstrated in previous studies (35, 40). In a phase 2 trial evaluating the efficacy of pembrolizumab in treating NSCLC patients with brain metastases, a brain metastasis response of 29.7% was observed in patients with PD-L1 expression of at least 1%, while there was no response in another cohort with PD-L1 expression <1% or unevaluable (35). However, due to the distinct immune microenvironment of brain metastases, the expression profile of PD-L1 can be pretty heterogenous between primary tumor sites and metastases, demonstrating both temporal and spatial discordance (58, 59). Therefore, although PD-L1 expression may work as a prognostic factor, the response rates of brain metastases can be pretty different from the primary tumor, and while guiding clinical decisions based on PD-L1 expression, biopsy acquisition from metastatic sites should be considered.

Several limitations in this meta-analysis should be acknowledged. First, the number of studies included in this meta-analysis is relatively small. Therefore, the conclusion is preliminary and should be cautiously interpreted, especially for those in subgroup analysis as some subgroups only contain one eligible study. Also, subgroup analysis based on the treatment line was not performed due to insufficient included studies in this meta-analysis. However, we should notice that patients receiving ICIs can be heavily pretreated in real-world clinical practice, and efficacy of immunotherapy is dependent on the line of treatment (10, 60). Second, all the included studies are *post-hoc* exploratory analyses with risk of bias to some extent, as inevitable imbalance of confounding factors presenting between treatment and control arms. Besides, most ongoing and completed clinical trials do not report survival outcomes of patients with specific metastatic sites. Thus, there may be a selection bias to some extent. Up to date, several clinical trials are ongoing investigating ICIs in solid tumor with brain metastases (52). Further investigations are warranted to elucidate organ-specific tumor immune microenvironment, and more randomized trials are required to compare the efficacy of immunotherapy with conventional therapy based on metastatic sites. Precise prognostic biomarkers of organ-specific response should also be identified to guide optimal clinical decisions.

## Conclusion

In conclusion, current evidence suggests that ICIs can significantly prolong OS in NSCLC patients with brain metastases, and both OS and PFS in those with liver metastases. Although brain and liver metastases are generally regarded as poor prognostic factors for immunotherapy, this study still indicates ICIs are effective therapeutic options for NSCLC patients with these metastatic sites.

## Data Availability Statement

All datasets generated for this study are included in the article/Supplementary Material.

## Author Contributions

LZ, JL, and KY: conceptualization. JL and KY: data curation and original draft writing. KY: statistical analysis. LZ, JL, KY, ZS, and CB: manuscript review and editing. All authors contributed to the article and approved the submitted version.

## Conflict of Interest

The authors declare that the research was conducted in the absence of any commercial or financial relationships that could be construed as a potential conflict of interest.
