# Supplementary material for: Efficacy of Immune Checkpoint Inhibitors in Non-small-cell Lung Cancer Patients With Different Metastatic Sites: A Systematic Review and Meta-Analysis
Source: Front Oncol. 2020 Jul 9;10:1098. doi: 10.3389/fonc.2020.01098 (PMC7363957; doi:10.3389/fonc.2020.01098)
Supplement: Supplementary file 1 [file Data_Sheet_1.docx]

Supplementary Table 1 Search strategies for PubMed, Embase and Cochrane Library

| **Database** | **Keywords** |
| --- | --- |
| **PubMed** |  |
| #1 | NSCLC[tiab] |
| #2 | "non-small cell lung cancer"[tiab] |
| #3 | lung*[tiab] OR pulmonary[tiab] OR bronchus[tiab] OR brochogenic[tiab] OR bronchial[tiab] OR bronchoalveolar[tiab] OR alveolar[tiab] |
| #4 | carcinoma*[tiab] OR cancer*[tiab] OR adenocarcinoma*[tiab] OR malignan*[tiab] OR tumor*[tiab] OR tumour*[tiab] OR neoplasm*[tiab] |
| #5 | non[tiab] AND small[tiab] AND cell[tiab] |
| #6 | "Carcinoma, Non-Small-Cell Lung"[Mesh] |
| #7 | #3 AND #4 AND #5 |
| #8 | #1 OR #2 OR #6 OR #7 |
| #9 | "Neoplasm Metastasis"[Mesh] |
| #10 | metastas*[tiab] |
| #11 | brain[tiab] OR cerebral[tiab] OR "central nervous system"[tiab] OR liver[tiab] OR bone[tiab] |
| #12 | #9 OR #10 |
| #13 | #11 AND #12 |
| #14 | checkpoint*[tiab] OR "checkpoint inhibitor"[tiab] OR CTLA-4[tiab] OR PD-1[tiab] OR PD-L1[tiab] OR ipilimumab[tiab] OR atezolizumab[tiab] OR durvalumab[tiab] OR pembrolizumab[tiab] OR nivolumab[tiab] OR avelumab[tiab] OR tremelimumab[tiab] |
| #15 | (#8 AND #13 AND #14) Filters: published in the last 10 years |
| **Embase** |  |
| #1 | 'non small cell lung cancer'/exp |
| #2 | 'metastasis'/exp |
| #3 | 'non-small cell lung cancer':ab,ti |
| #4 | 'nsclc':ab,ti |
| #5 | 'lung*':ab,ti OR 'pulmonary':ab,ti OR 'bronchus':ab,ti OR 'brochogenic':ab,ti OR 'bronchial':ab,ti OR 'bronchoalveolar':ab,ti OR 'alveolar':ab,ti |
| #6 | 'carcinoma':ab,ti OR 'cancer*':ab,ti OR 'adenocarcinoma*':ab,ti OR 'malignan*':ab,ti OR 'tumor*':ab,ti OR 'tumour*':ab,ti OR 'neoplasm*':ab,ti |
| #7 | 'non':ab,ti AND 'small':ab,ti AND 'cell':ab,ti |
| #8 | #5 AND #6 AND #7 |
| #9 | #1 OR #3 OR #4 OR #8 |
| #10 | 'metastas*':ab,ti |
| #11 | #2 OR #10 |
| #12 | 'brain':ab,ti OR 'cerebral':ab,ti OR 'central nervous system':ab,ti OR 'liver':ab,ti OR 'bone':ab,ti |
| #13 | #12 AND #11 |
| #14 | 'checkpoint*':ab,ti OR 'checkpoint inhibitor':ab,ti OR 'ctla-4':ab,ti OR 'pd-1':ab,ti OR 'pd-l1':ab,ti OR 'ipilimumab':ab,ti OR 'atezolizumab':ab,ti OR 'durvalumab':ab,ti OR 'pembrolizumab':ab,ti OR 'nivolumab':ab,ti OR 'avelumab':ab,ti OR 'tremelimumab':ab,ti |
| #15 | #9 AND #13 AND #14 |
| #16 | #15 AND ('clinical trial'/de OR 'cohort analysis'/de OR 'controlled clinical trial'/de OR 'prospective study'/de OR 'randomized controlled trial'/de) |
| **Cochrane Library** |  |
| #1 | MeSH: Carcinoma, Non-Small-Cell Lung |
| #2 | (non-small cell lung cancer):ti,ab,kw |
| #3 | (lung*):ti,ab,kw OR (pulmonary):ti,ab,kw OR (bronchus):ti,ab,kw OR (brochogenic):ti,ab,kw OR (bronchial):ti,ab,kw OR (bronchoalveolar):ti,ab,kw OR (alveolar):ti,ab,kw |
| #4 | (carcinoma):ti,ab,kw OR (cancer*):ti,ab,kw OR (adenocarcinoma*):ti,ab,kw OR (malignan*):ti,ab,kw OR (tumor*):ti,ab,kw OR (tumour*):ti,ab,kw OR (neoplasm*):ti,ab,kw |
| #5 | (NSCLC):ti,ab,kw |
| #6 | (non):ti,ab,kw AND (small):ti,ab,kw AND (cell):ti,ab,kw |
| #7 | #3 AND #4 AND #6 |
| #8 | #1 OR #2 OR #5 OR #7 |
| #9 | MeSH: Neoplasm Metastasis |
| #10 | (metastas*):ti,ab,kw |
| #11 | (brain):ti,ab,kw OR (cerebral):ti,ab,kw OR (central nervous system):ti,ab,kw OR (liver):ti,ab,kw OR (bone):ti,ab,kw OR (lung*):ti,ab,kw OR (pulmonary):ti,ab,kw |
| #12 | #9 OR #10 |
| #13 | #11 AND #12 |
| #14 | (checkpoint):ti,ab,kw OR (checkpoint inhibitor):ti,ab,kw OR (CTLA-4):ti,ab,kw OR (PD-1):ti,ab,kw OR (PD-L1):ti,ab,kw OR (ipilimumab):ti,ab,kw OR (atezolizumab):ti,ab,kw OR (durvalumab):ti,ab,kw OR (pembrolizumab):ti,ab,kw OR (nivolumab):ti,ab,kw OR (avelumab):ti,ab,kw OR (tremelimumab):ti,ab,kw |
| #15 | #8 AND #13 AND #14 |


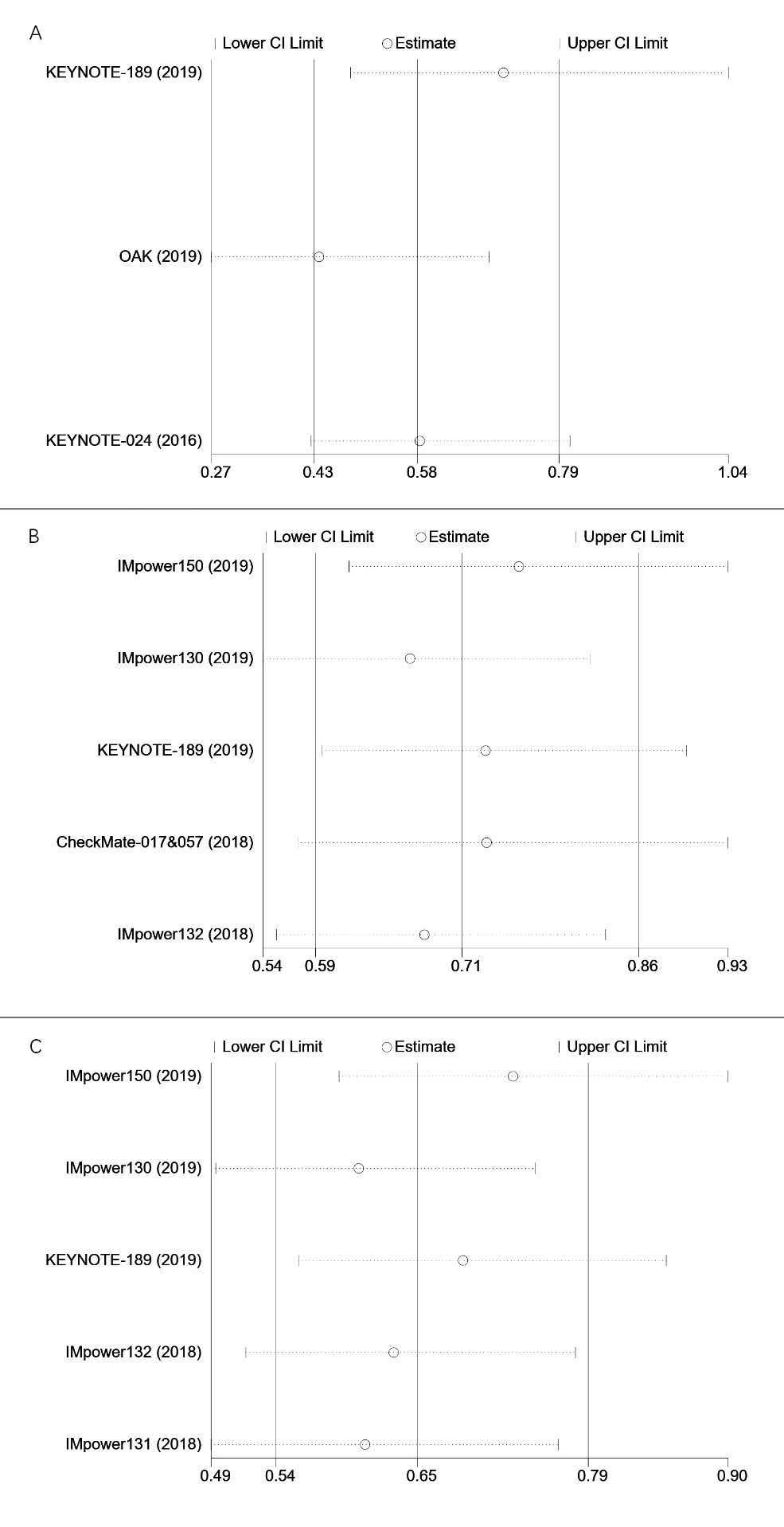


Supplementary Figure 1 Sensitivity analysis of (A) OS of patients with brain metastases (B) OS of patients with liver metastases (C) PFS of patients with liver metastases


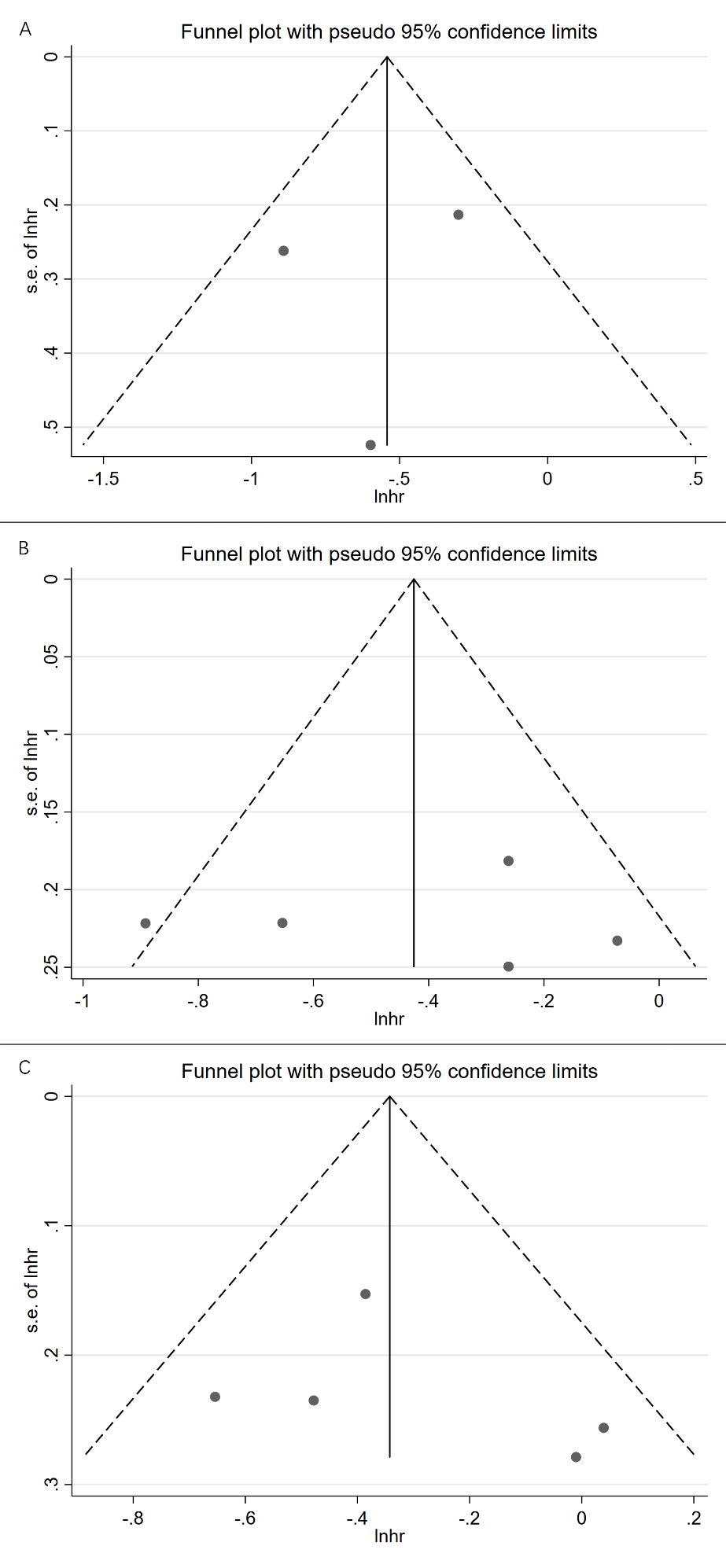


Supplementary Figure 2 Publication bias for (A) OS of patients with brain metastases (B) OS of patients with liver metastases (C) PFS of patients with liver metastases
